# Learning Grasp Configuration Through Object-Specific Hand Primitives for Posture Planning of Anthropomorphic Hands

**DOI:** 10.3389/fnbot.2021.740262

**Published:** 2021-09-15

**Authors:** Bingchen Liu, Li Jiang, Shaowei Fan, Jinghui Dai

**Affiliations:** State Key Laboratory of Robotics and Systems, Harbin Institute of Technology, Harbin, China

**Keywords:** grasping, anthropomorphic hand, postural synergy, dimension reduction, Gaussian mixture regression

## Abstract

The proposal of postural synergy theory has provided a new approach to solve the problem of controlling anthropomorphic hands with multiple degrees of freedom. However, generating the grasp configuration for new tasks in this context remains challenging. This study proposes a method to learn grasp configuration according to the shape of the object by using postural synergy theory. By referring to past research, an experimental paradigm is first designed that enables the grasping of 50 typical objects in grasping and operational tasks. The angles of the finger joints of 10 subjects were then recorded when performing these tasks. Following this, four hand primitives were extracted by using principal component analysis, and a low-dimensional synergy subspace was established. The problem of planning the trajectories of the joints was thus transformed into that of determining the synergy input for trajectory planning in low-dimensional space. The average synergy inputs for the trajectories of each task were obtained through the Gaussian mixture regression, and several Gaussian processes were trained to infer the inputs trajectories of a given shape descriptor for similar tasks. Finally, the feasibility of the proposed method was verified by simulations involving the generation of grasp configurations for a prosthetic hand control. The error in the reconstructed posture was compared with those obtained by using postural synergies in past work. The results show that the proposed method can realize movements similar to those of the human hand during grasping actions, and its range of use can be extended from simple grasping tasks to complex operational tasks.

## Introduction

Recent technological advances in robotics and related areas have led to the development of sophisticated anthropomorphic hands with an increasing number of degrees of freedom (DoFs) (Belter et al., 2013; Portnova-Fahreeva et al., 2020). Due to improvements in their flexibility, such anthropomorphic hands can perform tasks requiring dexterity in several areas of the manufacturing and services industries (Leidner et al., 2015). However, controlling an arm with several DOFs is a difficult task in which the accuracy of motion of all joints needs to be guaranteed and their trajectories need to be pre-planned according to the requirements of the given task (Shimoga, 1996; Bicchi and Kumar, 2000). For an industrial manipulator, an inverse kinematic technique is used to compute robotic joint motions, but this method requires a large amount of calculation (Chattaraj et al., 2014). In prosthetics, several grasping modes are planned in advance, the operator's intention is identified, and finite-state machines (FSM) or pattern recognition (PR) methods are used to switch between modes (Purushothaman, 2016). The disadvantages of this method are a slow controller and poor universality because different modes are discrete (Pylatiuk et al., 2007). It is thus important to find an appropriate control strategy to improve the operational capability and range of application scenarios of anthropomorphic hands.

The human hand is a complex system of 19 articulations, 31 muscles, and more than 23 DoFs. It can complete with ease a diversity of grasping tasks that require dexterous manipulation (Terlemez et al., 2015; Mandery et al., 2016). Improving our understanding of the human grasp control strategy can help simplify artificial manipulator control systems. Research in neuroscience has shown that the central nervous system can cope with redundant DoFs in a control space of reduced dimensionality (Mussaivaldi, 1999; Philippe et al., 2001; Philipp et al., 2016). Synergies are defined as the principal patterns of motor control in this context (Marco et al., 2013). By combining pre-organized patterns, the central nervous system can generate a variety of movements by simultaneously activating multiple DoFs, instead of separately controlling individual joints or muscles (Bian et al., 2012; Marques et al., 2014). Postural synergies were proposed in 1998 during the analysis of static grasping postures (Santello et al., 1998). In the relevant study, the authors recorded 15 angular positions of five subjects while they grasped 57 kinds of imagined objects. The results of principal component analysis (PCA) subsequently revealed that two main principal components (called postural synergies herein) accounted for more than 80% of the requisite postural information.

The theory of postural synergy was subsequently applied to the control of anthropomorphic hands. Ciocarlie and Allen (2009) proposed a grasp planner in a subspace of hand postures with highly reduced dimensionality, and performed posture reproduction on four models of hands through joint-to-joint mapping. Matrone et al. (2010) collected sensory data from a 16-DoF under actuated prosthetic hand while it performed 50 grasping tasks, and then used a PCA-based algorithm to drive the hand with a two-dimensional (2D) control input. Wimböck et al. (2011) proposed a synergy impedance controller for DLR Hand II. For torque-controlled robot hands, it can imitate the behavior of a synergistic under actuated hand. Its performance was verified for configurations of the hand as it held a ball and grasped a bottle. Bernardino et al. (2013) proposed a method to generate different types of precision grasps by using postural synergies extracted from data on the movement of the Shadow Hand and iCub Hand as they performed grasping tasks on 12 objects. Bicchi's group (Santina et al., 2018) designed Pisa/IIT SoftHand 2 based on the soft synergy model. This hand has only two degrees of actuation (DoAs) but can carry out a large variety of grasping and manipulation tasks by relying on the intelligence embodied in the mechanism (Piazza et al., 2020).

Although the ability of postural synergy to simplify control systems has been verified, most relevant research has focused on the reproduction of tasks already recorded in the relevant databases. It is challenging to generate the grasp configuration for a new task by using postural synergy. This problem can be solved by inferring the synergy coordinates of objects from basic shape descriptions of them (Rodriguez and Behnke, 2018; Rodriguez et al., 2018).

This study proposes a method to generate grasp configurations based on synergies of the hand and a shape descriptor. A dataset of human grasping actions was first created. Data on the movements of the hands of 10 subjects were recorded while they performed grasping tasks on five typical categories of objects. The sizes of the objects along the direction in which they were grasped were also recorded. Following this, four hand synergies were extracted by using the PCA method, and the trajectories of the corresponding average synergy inputs of each task were obtained through the GMM/GMR method. Gaussian processes (GPs) were then trained for each category of grasping task to infer the synergistic input trajectories of a given shape descriptor. Finally, the feasibility of the proposed method was verified by simulations on grasp configurations of a prosthetic hand control. The results showed that the proposed method can realize movements similar to those of the human hand during grasping actions, and its use can be extended to complex operational tasks.

The remainder of this paper is organized as follows: Section Experiment describes the creation of the database of human grasping tasks, and Section Method provides the method used to extract hand primitives and generate synergistic input trajectories. Section Result details the evaluation of the performance of the proposed method, and Section Discussion discusses the differences between its results and those of methods proposed in previous studies. Section Conclusion summarizes the conclusions of this study and suggests directions for future work in the area.

## Experiment

### Participants

Ten healthy subjects (22–28 years old; nine men and one woman, all of whom were right-handed) volunteered to participate in the experiment. All participants were in good health, and reported no history of neurological or motor disorders. We analyzed their preferences for pre-grasping shapes of the hand. The experimental procedure was approved by the Institutional Review Board (IRB) of the Harbin Institute of Technology in P. R. China. Before the experiment, all subjects provided their informed consent, including agreeing to the aims and duration of the entire experiment and its procedure.

### Experimental Environment and Protocol

**Figure 1:**
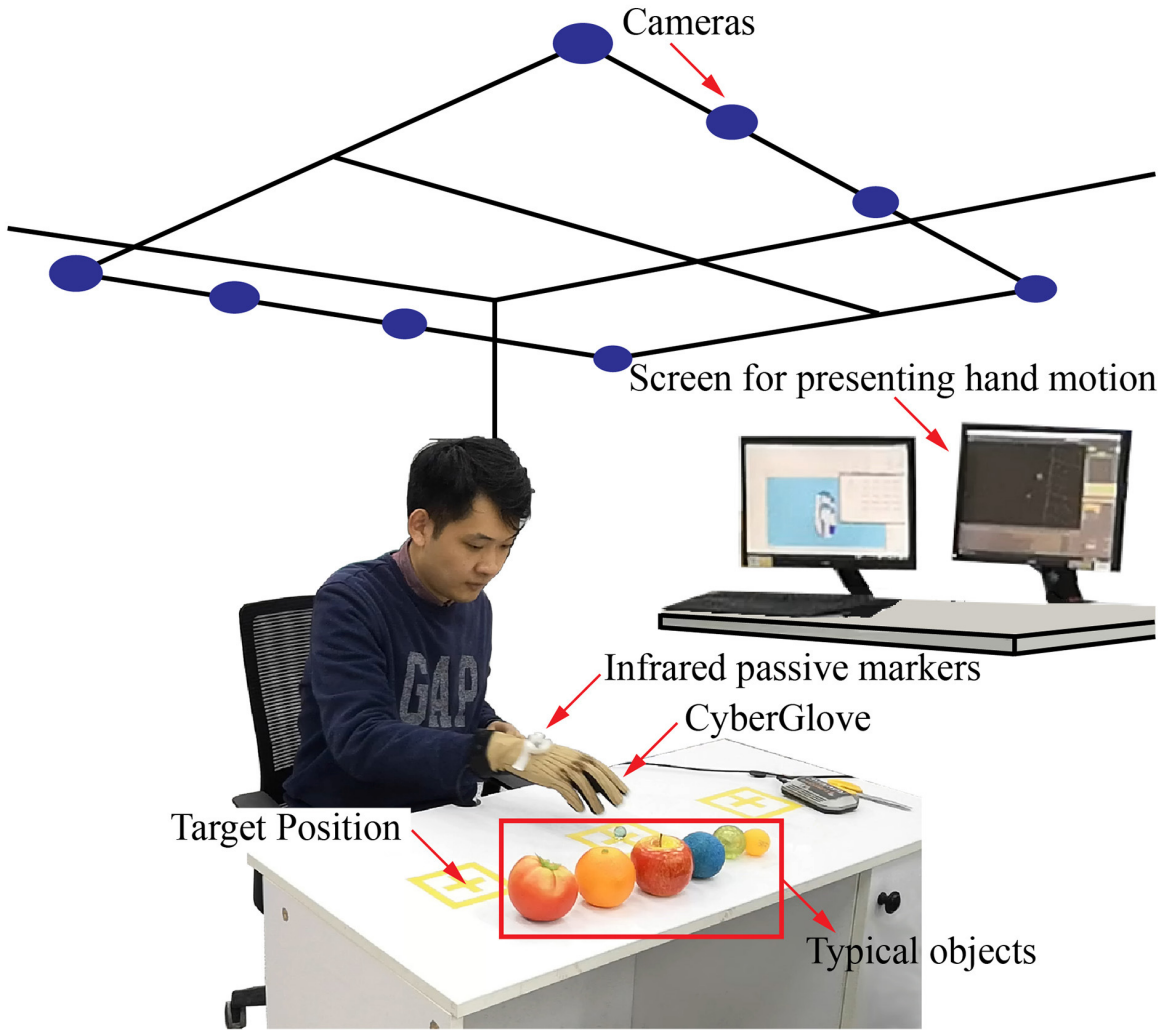
The experimental setup and the motion capture system.

**Figure 2:**
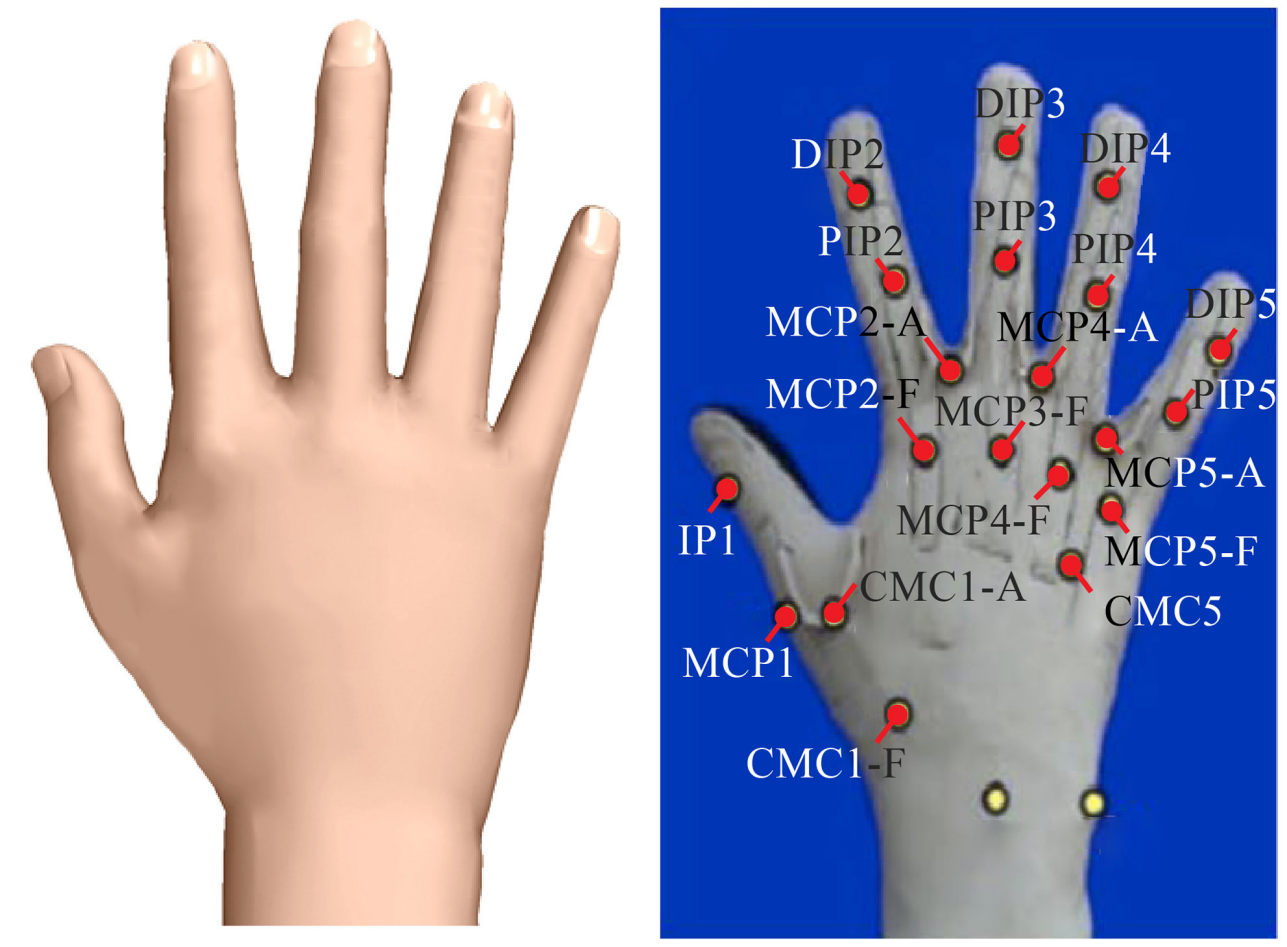
The angular data recorded by the Cyberglove. In total of 19 joints angular data containing flexion of the MetaCarpal-Phalangeal (MCP), Proximal Inter-Phalangeal (PIP), Distal Inter-Phalangeal (DIP) joints of the four fingers, the Carpometacarpal (CMC), MCP, and Inter-Phalangeal (IP) joint of the thumb, and the abduction joint (ABD) joints between adjacent fingers were recorded.

The experimental setup and equipments are shown in Figure 1. The experimental setup consisted of a visual tracking system, a Cyberglove, infrared passive markers (IRm), six sets of fundamental objects, and two reference target positions. CyberGlove (Virtual Technologies, Palo Alto, CA, USA) was used to measure the angular data on the joints during reach-to-grasp movements. As is shown in Figure 2, angular data on a total of 19 joints were recorded. A simple method to calibrate the joint angles was applied according to the linear mapping of their individual ranges of motion (Jarrassé et al., 2014).

The Vicon motion capture system (Oxford Metrics Ltd., Oxford, UK) was used in this study owing to its high accuracy (0.1 mm). The system comprised eight T-40s Vicon cameras to collect frames at 50 Hz. To capture the position and posture of the entire hand as well as the relative relationship between it and the object, three 9.5-mm reflective markers were placed on the back of each subject's dominant hand. Although we focused on hand postures here, the relationship between the hand and the object was also examined for future research.

**Figure 3:**
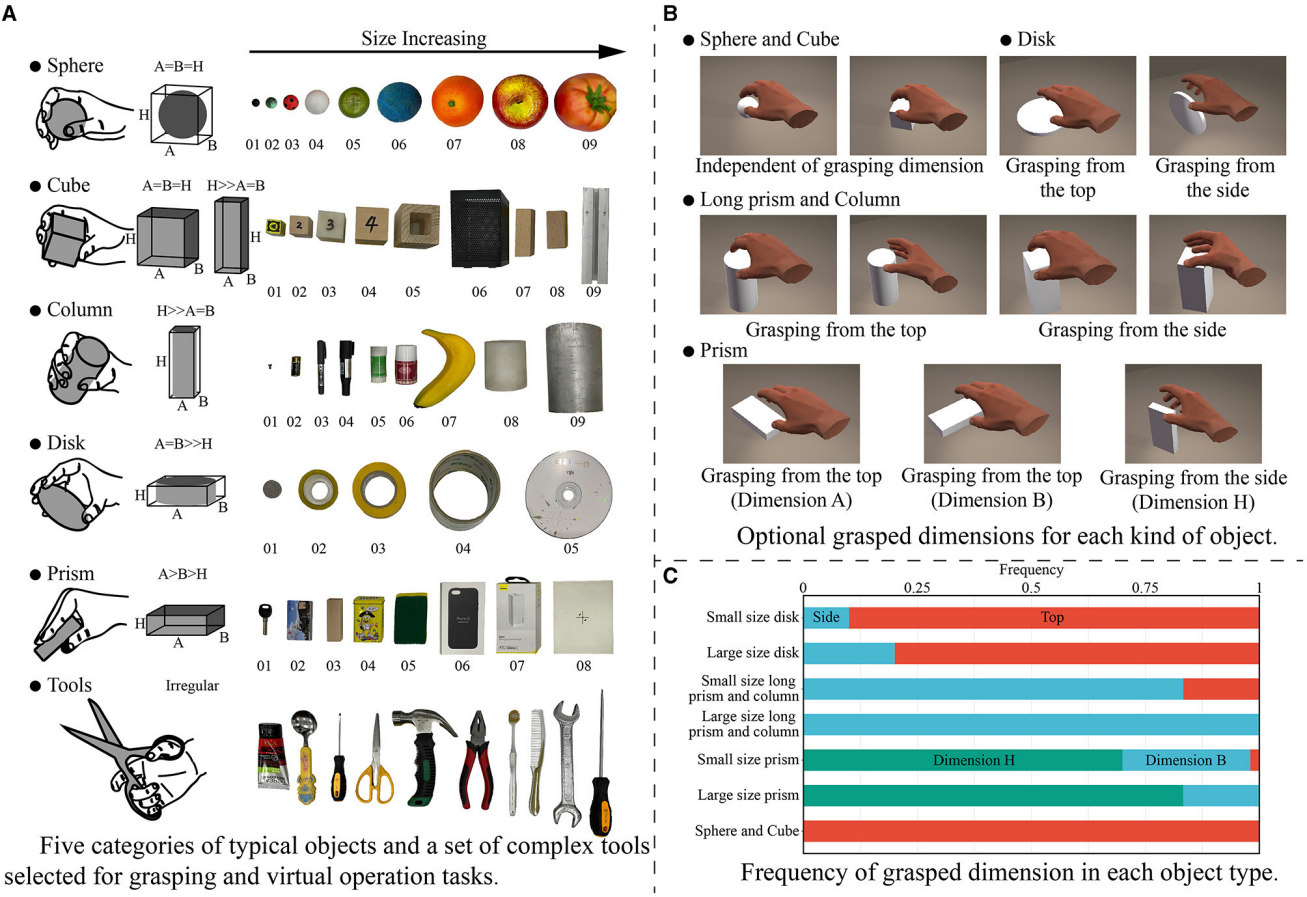
Five categories of typical objects and a set of complex tools used in this paper, and the type of grasping posture selected corresponding to the objects. **(A)** Five categories of typical objects and a set of complex tools selected for grasping and virtual operation tasks. **(B)** Optional grasped dimensions for each kind of object. **(C)** Frequency of grasped dimension in each object type.

The objects were chosen by referring to the YCB Object Set (Calli et al., 2015) and a method to categorize convex objects (Benn and Ballantyne, 1993). Finally, five categories of typical objects (sphere, cube, cylinder, disk, and cuboid) and a set of complex tools were selected. These objects with simple shapes were parameterized by the three primary axes of a, b, and h. The selected objects are shown in Figure 3A, and their dimensions are given in the Appendix.

The experiment involved two kinds of tasks. For typical objects, the subjects performed simple grasping and moving tasks. Before the experiment, the grasping habits of each subject were investigated. The approach direction and orientation for each kind of object were considered, and the possible grasped dimensions were listed as shown in Figure 3B. The subjects were asked to select the direction from which they thought could achieve stably grasping, and the frequency of selected posture across subjects were shown in Figure 3C. The subjects had a clear tendency to grasp objects along the side with the smallest dimension except in case of disks, but there were some differences for individual objects. For Disk01 and Disk05, several subjects thought that grasping from the side was more conducive to the stability of grasping. For Prism04 and Prism04, a part of subjects chose to grasp from the secondary short dimension, because it was more consistent with the hand natural aperture. While for Column05 and Column06, some subjects selected to pick up the object from the top in line with their grasping habits. Finally, the grasping posture for each kind of object was unified to avoid large differences in the same tasks. Long objects were selected to grasp from the side, rather than using the longest dimension. Disks were formulated to grasp from the top, whereas prisms were defined to grasp by the smallest dimension. The final grasping posture was consistent with those used in previous studies (Feix et al., 2014).

Once they had familiarized themselves with their grasping postures for the objects, the subjects were asked to move each object from its initial position to a target position according to instructions on a desktop computer. The start command was issued by the computer, and the requisite grasping postures and task progress were shown on the screen. With tasks requiring tools, subjects needed to perform virtual operations according to the functions of the given tool. In these tasks, the subject needed to adjust to a firm grasping posture after picking up the object, and then performed schematic operations based on the object's characteristics and function. The operational data enabled us to better express the operational space of the hand, and determine whether there was a difference between the human instinctive reaction and the given operational purpose. These data can also help extend traditional research on static grasping postures.

The subjects were required to perform each grasping and moving or virtual operation task 10 times.

## Methods

**Figure 4:**
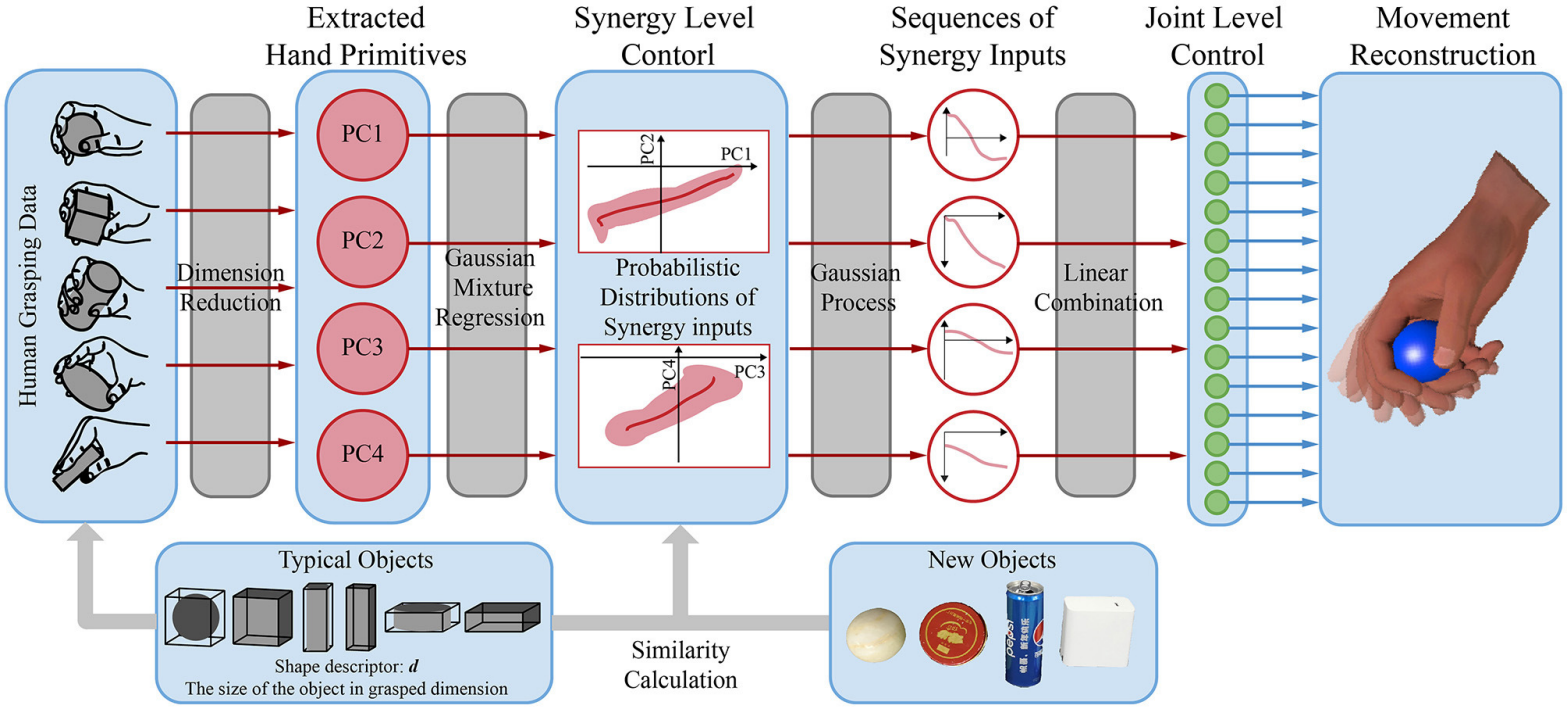
The framework of extracting object-specific hand primitives and the synergy inputs for trajectories according to the size of the object.

Figure 4 shows the framework used to extract temporal synergic strategies according to the size of the object. Principal component analysis (PCA) was first applied to the data on grasping to establish a low-dimensional synergy space and obtain hand primitives. Gaussian mixture regression (GMR) was then used to reconstruct a unique and dynamic model for each object sample. Following this, several GPs were trained to generate trajectories at the synergistic level by using the similarity between objects. Finally, the trajectories in the synergistic space were mapped back to the joint space to obtain a sequence of grasping postures. The implementation is explained below.

### Dimension Reduction and Hand Primitives Extraction

PCA can be used to analyze the synergies of hand pstures (Patel and Burns, 2015). Linear hand primitives (called postural synergies) extracted by PCA are highly interpretable, and can be easily reproduced by a mechanical structure (Brown and Asada, 2007). Therefore, we used the PCA algorithm to obtain hand primitives in this study.

A second-order two-way low-pass Butterworth filter was applied on the data on the joint angles at a cut-off frequency of 5 Hz. To ensure that all grasping movements made by each subject had the same weight, the angles of each of their joints were normalized to the range [0, 1], according to the minimum and maximum values of the sensor data obtained in the calibration process. Owing to the poor accuracy of DIP joint measurement in some subjects, the DIP angular data were discarded altogether. Finally, the posture matrix ***J*** that was obtained contained postures from 10 subjects when performing 50 kinds of grasping movements:


(1) $$\begin{array}{l} J\;=\;\left[j_{1};\;j_{2};...;j_{N}\;\right]\in {ℜ}^{N\times D} \end{array}$$

where *j* ∈ ℜ^1 × *D*^ represents a hand posture vector, *D* was 15 according to the number of preserved joint angles, and *N* is the number of sample posture vectors recorded in the dataset.

The hand primitives can be calculated as eigenvectors $s_{i}\in {ℜ}^{15\times 1}\left(i<15\right)$ of the covariance matrix of the posture matrix $\tilde{j}$. Each posture data was zero-centered by being reduced to the average posture ${\tilde{j}}_{i}=j_{i}-\frac{1}{N}\sum_{i-1}^{N}j_{i}$. Then, the covariance matrix $\Sigma \;=\;\frac{{\tilde{j}\tilde{j}}^{T}}{N}$ of the zero-centered posture matrix was calculated and decomposed as follows:


(2) $$\begin{array}{l} \Sigma =SES^{T} \end{array}$$

Where *E* = *diag* (δ_1_, δ_2_, …, δ_*D*_) is the matrix of eigenvalues and *S* = [*s*_1_ *s*_2_…*s*_*D*_] is the eigenvector matrix. Finally, eigenvectors corresponding to the *k* largest eigenvalues were reserved as the hand primitives.

Each hand posture was thus approximated as a linear combination of these hand primitives:


(3) $$\begin{array}{l} {{\tilde{j}}_{i}}^{T}={\bar{j}}_{i}^{T}+\;{\left[S_{1}...S_{k}\;\right]}_{15\times k}{\left[\begin{array}{c} W_{1}\\ \vdots \\ W_{k} \end{array}\right]}_{k\times 1} \end{array}$$

where $S=\left[s_{1}\;s_{2}\ldots s_{k}\right]\in {ℜ}^{15\times k}$ represents the matrix of hand primitives, and is time invariant. The vector $w_{i}=\left[w_{1};w_{2};\ldots \;;w_{k}\right]\in {ℜ}^{k\times 1}$ represents the synergy inputs, and is the projection of original data on the eigenvectors. For continuous movement, it can be regarded as a sequence of synergy input vectors controlling the hand primitives. Therefore, if the synergy input sequence is obtained, the grasping movement can be dynamically reproduced for each task.

### Gaussian Mixture Regression of Grasps

For each task, several steps of data preprocessing were applied to the synergy input sequence ***W***:


(4) $$\begin{array}{l} W={\left[w_{1}w_{2}\;\ldots w_{t}\right]}^{T}\in {ℜ}^{t\times k} \end{array}$$

For each trajectory, the parts before the start of the movements and after the object had been stably grasped were manually removed, and the remainder was resampled to 100 frames. The mean value of the synergy input sequence of each task was then calculated for each subject. In this way, 10 sets of sequences of synergy inputs $W_{i}\in {ℜ}^{100\times k}$ were obtained for each task.

To fit the probabilistic distributions of the joints to the sequences of synergy inputs, the Gaussian mixture model (GMM) was applied, and was defined as follows:


(5) $$\begin{array}{l} \left[\begin{array}{c} t\\ W \end{array}\right]\sim \overset{3}{\underset{n-1}{\sum }}\pi_{n}\;N\;\left(\mu_{n},\sum_{n}\right) \end{array}$$

where ***t*** represents the temporal value vector, and π_*n*_, μ_*n*_, and ∑_*n*_ represent the prior probability, the covariance and the mean of the *n*^*th*^ Gaussian component, respectively. The GMM was computed by initializing the mixture of Gaussian components with k-means clustering and optimizing them through expectation maximization. The number of Gaussian components was set to three because using more components did not improve the generalization capabilities of the latent trajectory model (Romero et al., 2010).

Following the above, Gaussian mixture regression (GMR) was applied to reconstruct a reference sequence of synergy inputs for each task (Calinon et al., 2007). For each Gaussian component *n* at given time step *t*, the estimated instantaneous mean and covariance of synergy input ***w*** are


(6) $$\begin{array}{l} {\overset{⌢}{\mu }}_{w,n}=\mu_{w,n}+\sum_{wt,n}{\left(\sum_{t,n}\right)}^{-1}\left(t_{n}-\mu_{t,n}\right)\\ {\sum^{⌢}}_{w,n}=\sum_{w,n}-\sum_{wt,n}{\left(\sum_{t,n}\right)}^{-1}\sum_{tw,n} \end{array}$$

As the result of a mixture of *n* Gaussian components, the conditional expectations of the mean and covariance of the synergy input ***w*** at a given time step *t* are


(7) $$\begin{array}{l} {\overset{⌢}{\mu }}_{w}=\sum_{n-1}^{3}\beta_{n}{\overset{⌢}{\mu }}_{w,n}\\ {\sum^{⌢}}_{w}=\sum_{n-1}^{3}\beta_{n}^{2}{\sum^{⌢}}_{w,n} \end{array}$$

where β_*n*_ is the probability that the Gaussian component *n* is responsible for time step *t*. Thus, by calculating $\left[{\overset{⌢}{\mu }}_{w},{\overset{⌢}{\sum }}_{w}\right]$ at different time steps *t*, a generalized form of the sequences of synergy inputs associated with the covariance matrices was generated.

### Learning Postural Synergies

The obtained sequences of synergy inputs can be used to generate postures for new movements under certain constraints. Humans can transfer their previous knowledge of similar objects to a new instance and generate an appropriate grasp. In light of this, we propose a supervised learning method to learn grasping movements according to the shape of the object. Previous studies have shown that the size of the object along the grasped dimension is closely related to grasping postures (Dessalene et al., 2019; Starke et al., 2020). Therefore, this was used as a shape descriptor to establish the relationship with grasping movements.

We encoded the shape descriptor and the sequences of synergy inputs with a GP-based machine learning method. We assumed that the synergy inputs associated with the same category of objects were distributed in a Gaussian manner. Thus, according to the number of synergy inputs, several GPs were trained (Rasmussen and Williams, 2004). All GPs were parametrized with the radial-basis function (RBF) kernel. The GPs encoded the desired relation between inputs ***D*** to the shape descriptor and the outputs ***W*** of the synergy trajectory as


(8) $$\begin{array}{l} \left[\begin{array}{c} W\\ w^{*} \end{array}\right]\sim \;N\;\left(\vec{0},\left[\begin{array}{cc} K\left(D,D\right)+\sigma_{n}^{2}I & K\left(D,d^{*}\right)\\ K\left(d^{*},D\right) & K\left(d^{*},d^{*}\right) \end{array}\right]\right) \end{array}$$

where *N* represents the Gaussian distribution, and w^*^ is the predicted synergistic trajectory corresponding to a new shape descriptor d^*^. The best predicted output is the mean value of the normal distribution.


(9) $$\begin{array}{l} w^{*}=K\left(d^{*},D\right){\left[K\left(D,D\right)+\sigma_{n}^{2}I\right]}^{-1}W \end{array}$$

Finally, the continuous movement of the hand is obtained by mapping the synergy input sequence back to the joint space as shown in equation (3). Through Gaussian regression, the temporal grasping postures for a new object can be constructed by using the hand primitives, instead of planning the motion of each joint independently for each task.

## Result

### Extracted Hand Primitives

**Figure 5:**
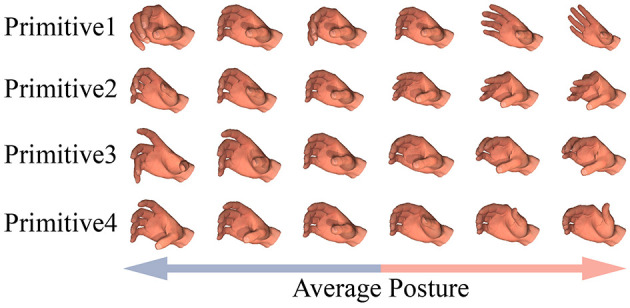
The contributions of four extracted hand primitives to the posture configuration.

Four PCs were extracted as hand primitives for the 10 subjects as they explained 87% of the variance. The contributions of these four primitives to the posture configuration are shown in Figure 5. The main effect of each hand primitive can be summarized as follows:

- Hand Primitive 1: This featured the flexion of MCP and PIP joints of the four fingers, and the range of bending increased gradually from the index finger to the little finger. This primitive controlled the overall posture of the hand, and had the most important influence on grasping.
- Hand Primitive 2: This consisted of the reverse movement of the MCP joints of the first four fingers and PIP joints of the last four fingers. In other words, when the MCP joints of the thumb, index, middle, and ring finger were flexed, the PIP joints of the index, middle, ring, and little finger were extended, and vice versa. This primitive was important for the overall configuration, especially when grasping cubic and prismatic objects.
- Hand Primitive 3: This consisted of the reverse movement of joints in the index, middle, ring, and little fingers. When the index finger approached the object, the middle, ring, and little fingers moved away from it, and vice versa. This primitive usually determines the form of applied force for the grasping task.
- Hand Primitive 4: This consisted of the adduction of the thumb and the four fingers. This primitive controlled the range of spreading of the entire hand. It also played an important role in the flexion movement of the thumb.

**Figure 6:**
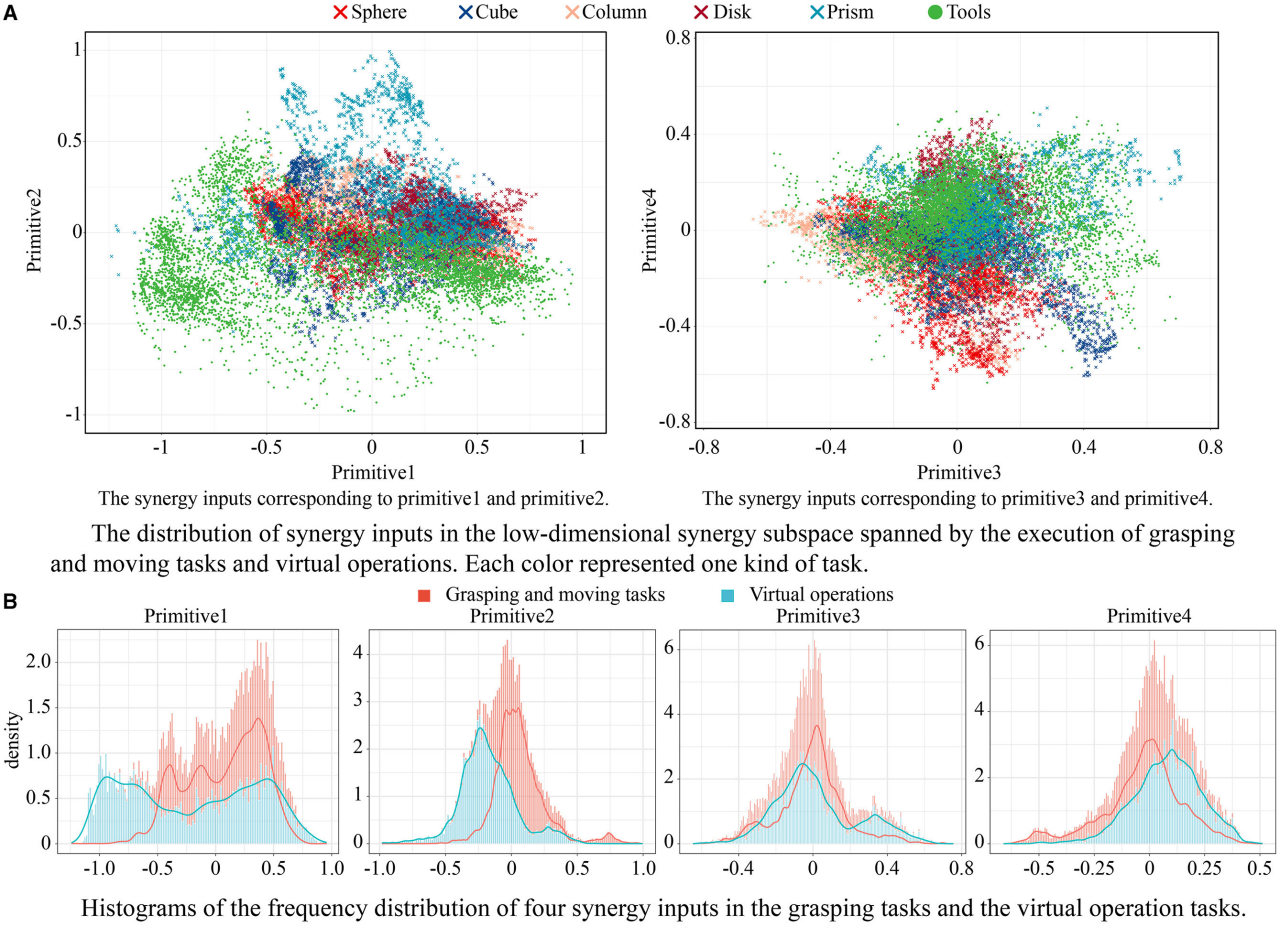
The values of the low-dimensional synergy input corresponding to the above four hand primitives, and the comparison results of frequency distribution of these hand primitives between grasping tasks and virtual operation tasks. **(A)** The distribution of synergy inputs in the low-dimensional synergy subspace spanned by the execution of grasping and moving tasks and virtual operations. Each color represented one kind of task. **(B)** Histograms of the frequency distribution of four synergy inputs in the grasping tasks and the virtual operation tasks.

The values of the low-dimensional synergy input corresponding to the above hand primitives are shown in Figure 6A. Similar trends can be observed for these categories of tasks. In the plane consisting of primitives 1 and 2, all the movements had a common starting point in the lower-right corner. In the plane consisting of primitives 3 and 4, the starting point was at the center. This shows that different movements had roughly the same starting posture, where primitive 1 was slightly lower than zero, and primitives 2, 3, and 4 were close to zero.

When the subject grasped a spherical, columnar, or disk-shaped object, the change in primitive 1 during the grasping movement was significant, while the range of values of primitive 2 was narrow. The trajectories of primitives 1 and 2 in these tasks presented a zonal distribution. When the subject grasped a cubic or a prismatic object, a significant variation in primitive 2 was observed for different objects of different sizes. The trajectories of primitives 1 and 2 in these tasks presented a fan-shaped distribution. The distributions of synergy inputs on the plane of primitives 3 and 4 were more similar for different tasks. The variation in primitive 4 was usually small, and primitive 3 moved toward the negative direction as the movement progressed. When the subject grasped a disk-shaped object, a large synergy input was recorded because the disk had a large diameter of 120 mm.

In addition, values of the synergy inputs for the virtual operation tasks involving tools covered a larger area in the synergy subspace than those of typical grasping tasks. This shows that the operational tasks offered a suitable complement to the hand movement database. Histograms of the frequency distribution of four synergy inputs in the grasping tasks and the virtual operation tasks are shown in Figure 6B. A majority of the values of primitive 1 for the operation tasks were significantly lower than those for the grasping tasks; primitive 2 showed the same trend. The distribution of primitives 3 and 4 in the operational tasks and the grasping tasks were consistent. The experiments showed that the subjects tended to use precision pinching instead of power grasping when picking up and moving the objects. Only when further operational tasks were posed did the subjects switch to the power grasping mode to firmly grasp the object in the palm. This phenomenon should be considered when planning postures for anthropomorphic hands.

**Figure 7:**
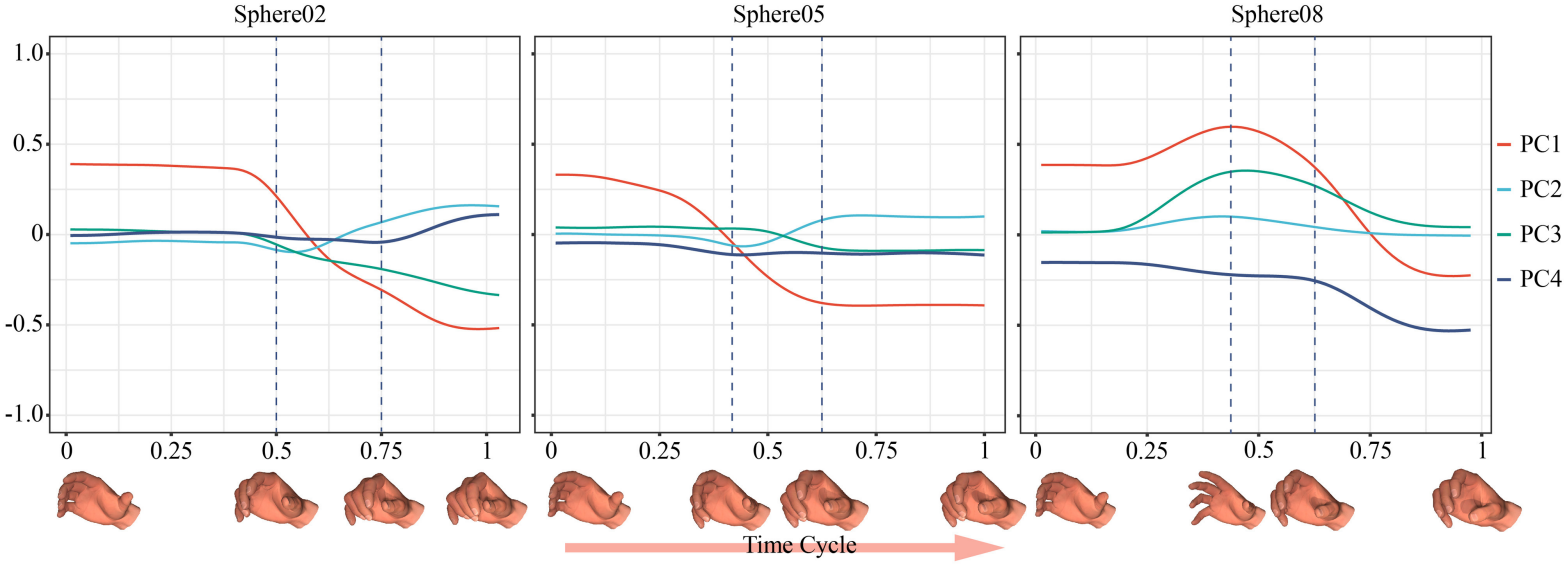
Samples of trajectories of the synergy inputs when grasping Sphere02, Sphere05, and Sphere08.

It was also important to record the trajectory of the entire movement. The fingers may not execute the flexion movement during the grasping task. In a previous experiment (Ong et al., 2019), the participants spread their fingers to increase contact with the object to stabilize the grasp. This phenomenon was verified in this paper. Figure 7 shows samples of trajectories of the synergy inputs when grasping spheres of three sizes. When grasping a larger sphere, the subjects opened their hands before performing the enveloping movement, which helped avoid premature collision and increased the contact area. If our system had considered only the final grasping posture, this strategy would have been difficult to identify.

### Generating Sequences of Synergy Inputs

**Figure 8:**
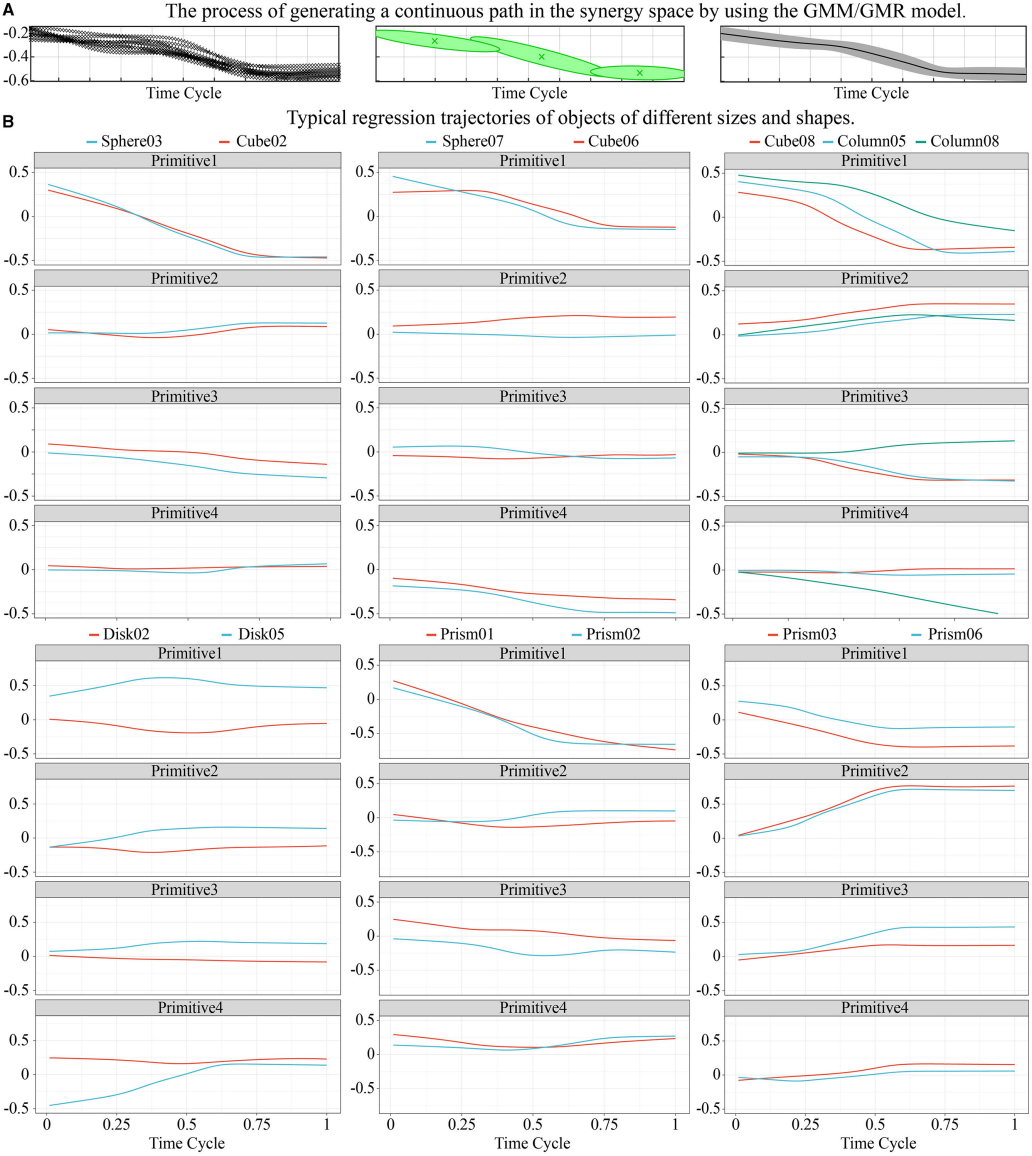
The process of generating a trajectory of the synergy input and several typical regression trajectories of objects of different sizes and shapes. **(A)** The process of generating a continuous path in the synergy by using the GMM/GMR model. **(B)** Typical regression trajectories of objects of different sizes and shapes.

Figure 8A shows the process of generating a continuous path in the synergy space by using the GMM/GMR model, where the green ellipses indicated the corresponding Gaussian components used to approximate the trajectory of the synergy input. The mean and variance of the regression trajectory are shown on the right of the figure. The results show that three Gaussian components yielded good regression performance.

**Figure 9:**
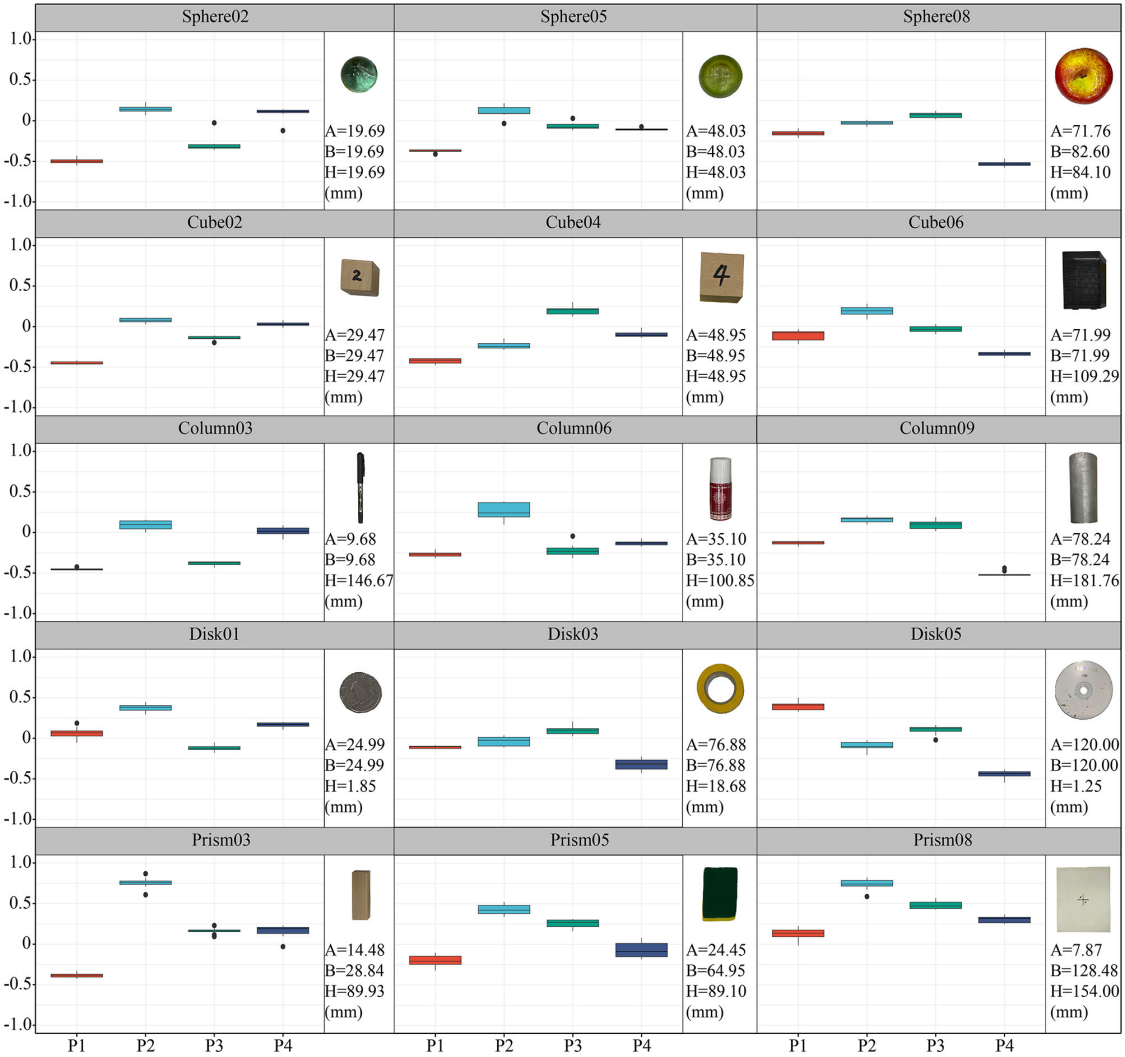
The synergy inputs on the frame of the final grasping postures for typical objects.

Figure 8B shows several typical regression trajectories of objects of different sizes and shapes. For the convenience of comparison, the synergy inputs on the frame of the final grasping postures for typical objects are also shown in Figure 9. The dynamic characteristics of trajectories of the synergy inputs of the four primitives and their relationships with the size of the object are now discussed by using the grasping of spherical objects as example.

**Figure 10:**
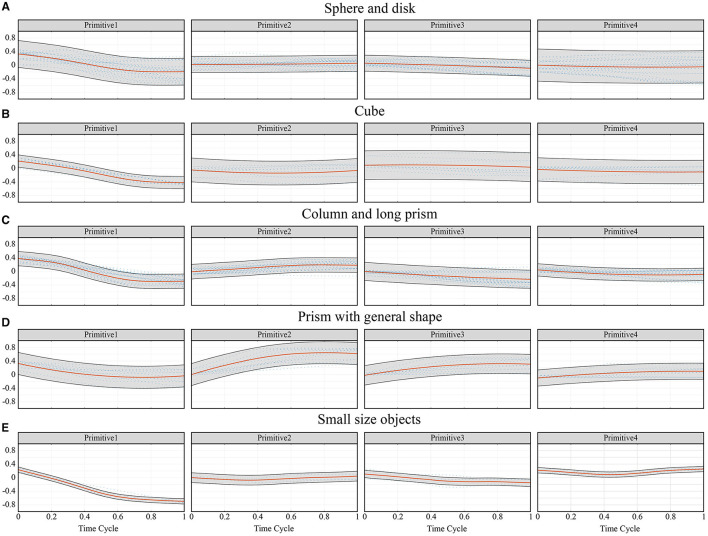
The trend of primitives for each category of objects inferred from the mean predictions of the Gaussian Processes. The red line represented the mean value, and the gray shaded area represented the covariance. **(A)** Sphere and disk, **(B)** Cube, **(C)** Column and long prism, **(D)** Prism with general shape, **(E)** Small size objects.

Primitives 1 and 4 had the highest correlation with the size of the object. With increasing object size, the value of primitive 1 gradually increased while that of primitive 4 gradually decreased. The posture gradually reduced the flexion of the last four fingers, and led the thumb to be adducted to the direction of the palm. This trend is clear in Figures 9, 10, and this rule was applicable to most tasks except the grasping of prism-shaped objects. On the whole, primitive 1 usually had the widest range of variation, and played a major role in the first half of the movement. This shows that people usually approach objects quickly at the beginning of grasping movements and then make adaptive adjustments.

Figures 8B, 9 show that the effect of primitive 2 was not prominent in grasping spherical objects, possibly because the main function of primitive 2 was to adjust the angle between the distal phalanx and the object. However, when grasping spherical objects, the fingers could wrap around the object well so that there was no need to adjust its value. The value of primitive 3 usually decreased with the size of the object, and contributed in the middle and later stages of the movement. The main function of primitive 3 was to control the range of bending of the last three fingers, but its effect was not as prominent as that of primitive 1. Therefore, this primitive was suitable for finely adjusting the tightness of the grip according to the size of the object, and its range of change for each task was small.

The grasping of prism-shaped objects was significantly different from the other tasks, as is clear from Figures 9, 10. This is because of different requirements for grasping oblate objects, which have been often ignored in previous studies. When only primitive 1 operated, the joints of all fingers turned in the same direction for the grasping action, and the fingers quickly approached the object. However, this caused the angle between the object and the finger to become too large or even vertical, which is not consistent with the scenario of a human hand grasping an object. The angle between the finger and the object determined the direction of the grasping force, and thus was important for ensuring the stability of grasping and the further enveloping of the object. Figure 8B shows that the role of primitive 2 in grasping prism-shaped objects was the most prominent. It influenced the first half of the movement, and was highly synchronized with primitive 1.

Prism01 and prism02 were different from movements for objects in the same category. They represented a lateral pinch for small, oblate objects. This kind of movement is frequent in daily life. Although the four fingers had a large flexion angle during the relevant motion, as when grasping spherical objects, the posture of the thumb was not similar. Therefore, it was necessary to divide these movements into separate kinds.

For categories of objects of similar sizes, the trajectories of the synergy inputs were often close. Figure 8B shows that the trajectories of Cube08 and Column05 were almost coincident. A similar strategy could be used for the precision grasping of long prism-shaped and cylindrical objects of similar sizes. The trajectories of small spheres and cubes were also very similar, such as sphere03 and cube02 in Figure 8B. However, as the size of the object increased, the curvature of its surface changed, and the corresponding requisite trajectories shifted. This phenomenon was most prominent in the trajectory of primitive 2, and led to a difference in grasping configuration between motions used to grasp cubic and spherical objects. Because a disk can be regarded as the cross-section of a sphere, the same strategy could be used for it as for grasping spherical objects.

To sum up, the grasping movements of five typical objects were re-divided according to the similarity in the trajectories of the synergy inputs. The following five categories of grasping strategies were determined as follows.

Category 1: Including spherical objects (A≈B≈H, with large surface curvature) and disk objects(A≈B>>H, with large curvature on cross section). The most widely used type of grasping posture, with a large distribution area of synergy inputs in primitive 1 and primitive 4.

Category 2: Including cube objects (A≈B≈H, with small surface curvature). Grasping strategy in this category was quite similar with that of Category 1, and the difference was distribution area of synergy inputs in primitive 2 was wider.

Category 3: Prolate objects including columns and long prisms (A≈B < < H). These objects were usually grasped from the side, with a large value in primitive 2 and low value in primitive 3.

Category 4: Including oblate objects whose convex hull could be regarded as a prism with general shape (A>>B>>H; A≈B>>H, with small curvature on cross section). These objects were usually grasped by the smallest dimension, with the highest value of primitive 2 and primitive 3.

Category 5: Objects with small volume and are very thin. These objects usually use lateral grasping posture.

The above five cases can basically include common objects in daily life. For objects with special shapes, their convex hull can be used to get an appropriate grasping strategy. In Figure 10, the synergy inputs inferred from the mean predictions of the Gaussian Processes for each category given the shape descriptor were shown. In the subsequent posture reconstruction experiment, similar grasping strategies were used for objects of the same category.

### Posture Reconstruction Experiment

**Figure 11:**
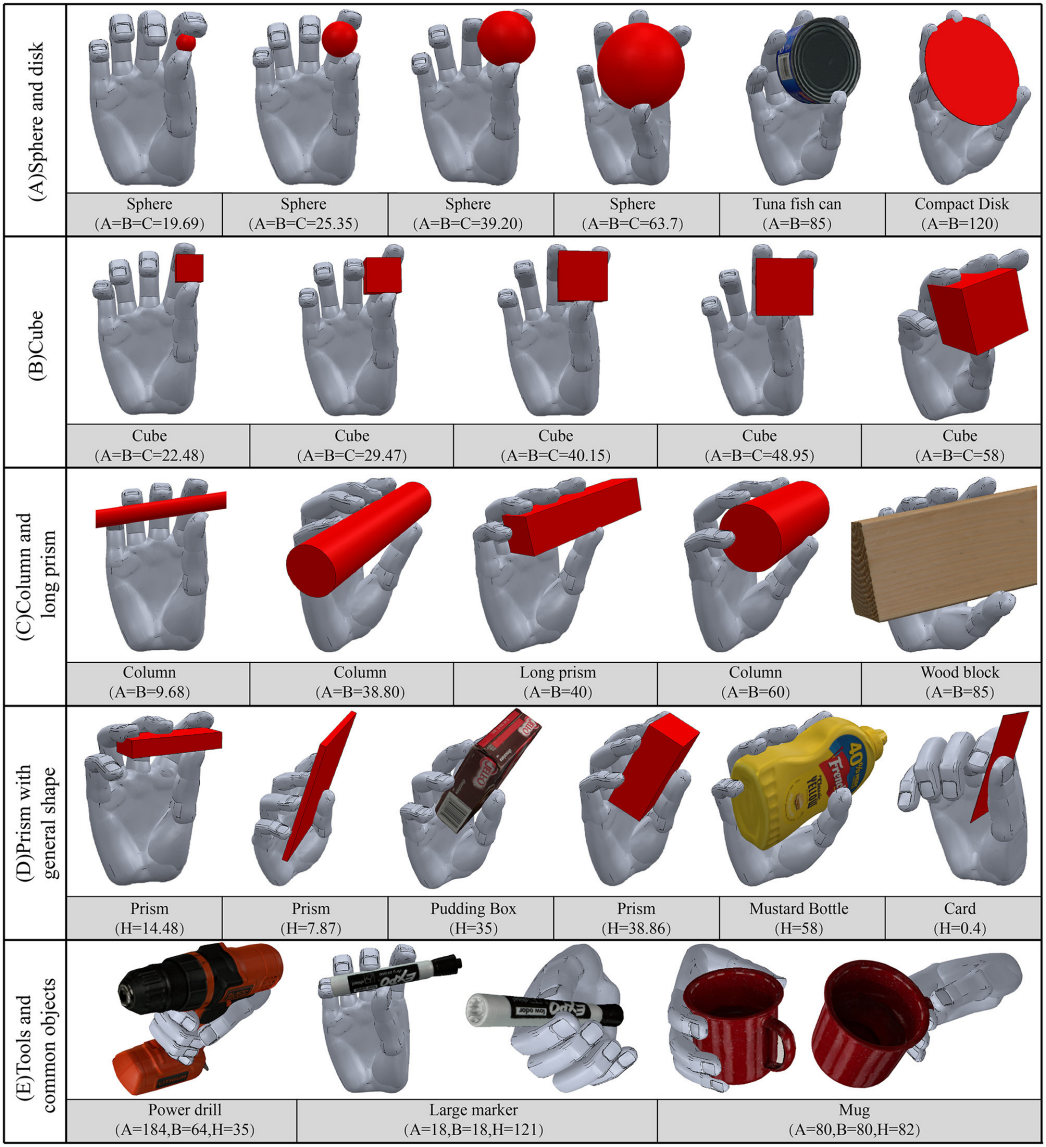
The results of virtual grasping experiment on typical objects.

To evaluate the performance of the proposed framework, the regressed trajectories were used to reproduce the grasping movements. A virtual grasping experiment was carried out by using a standard model of the human hand. The model featured four independent degrees of freedom in each finger, including one adduction joint and three flexion joints. Because DIP joint data were not available, we assumed that the ratio of the angular velocity of the DIP joint to that of the PIP joint was 2:3, and that the DIP joint stopped moving after coming into contact with the object. A simulation was carried out in V-REP(v3.3.2), and the result of posture reconstruction is shown in Figure 11.

Figure 11 shows changes in the grasping postures with the size of the objects, and six new objects in the YCB object set were introduced. For spheres and cubes, the grasping positions gradually moved from the index finger to the hollow of the palm with the increase in object size, and the thumb adducted to the palm. When grasping cubic and column-shaped objects, the four fingertips were nearly parallel to the objects and opposite the thumb. The MCP joints bent only slightly, which showed the function of primitive 2. When grasping a prism-shaped object, the four fingers were not exactly parallel to the object, and instead made a small acute angle with it. The thumb was roughly opposite the middle finger.

Figure 11 shows that the index finger did not come into contact with objects in some tasks, such as those involving the grasping of large spheres and squares. This is because none of the four groups of hand primitives was responsible for the independent movement of the index finger. However, when grasping large objects, the role of the index finger was not significant. In past work (Abbasi et al., 2016), large forces for the thumb, middle finger, and palm were recorded when grasping objects with large diameters, while a small force was recorded for the index finger. For tools object, the trajectories of the synergy inputs were planned independently. In order to perform corresponding tasks, the power drill should be firmly grasped in the palm. Therefore, the value of primitive 1 should be low as discussed in Extracted hand primitives. In addition, grasping postures were also closely related to task requirements. If only considering the shape of the object, the large marker and mug should be grasped from the side. However, when the constraints introduced by a task, the grasp choice also changed. The large marker should be held from the top, and the mug was always grasped from the handle.

**Figure 12:**
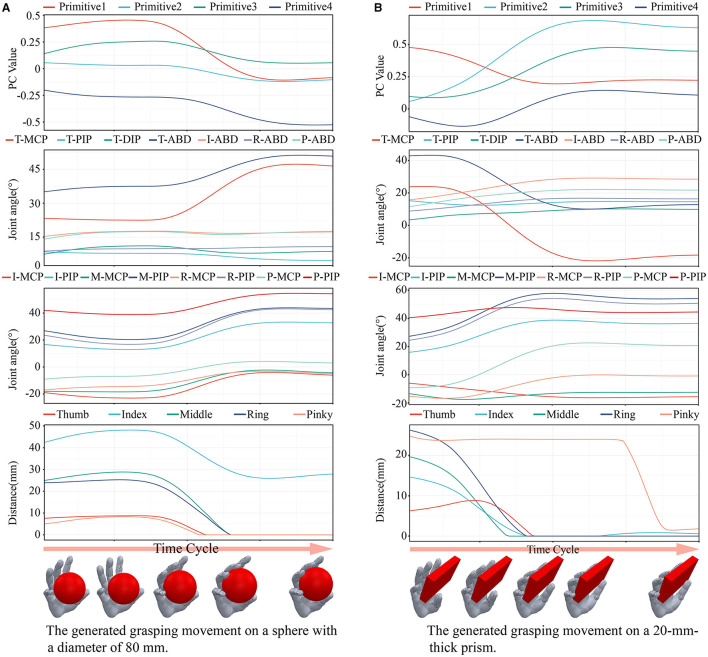
The generated grasping movements of two objects which were not included in the dataset above. **(A)** The generated grasping movement on a sphere with a diameter of 80 mm. **(B)** The generated grasping movement on a 20-mm-thick prism.

Figure 12 shows the generated grasping movements on a sphere with a diameter of 80 mm and a 20-mm-thick prism, respectively. These two objects were not included in the dataset above. From top to bottom are shown the trajectories of the synergy inputs, angular data of the joints, and the distance between the fingertip and the object. The figure shows that the trend of the reconstructed movement was the same as that in the grasping experiment involving humans. A greater adjustment space was obtained by spreading the palm, and the difference in contact frame between each finger and the object was not large. These results show that the generated trajectories of the synergy inputs met the requirements of the grasping task.

**Figure 13:**
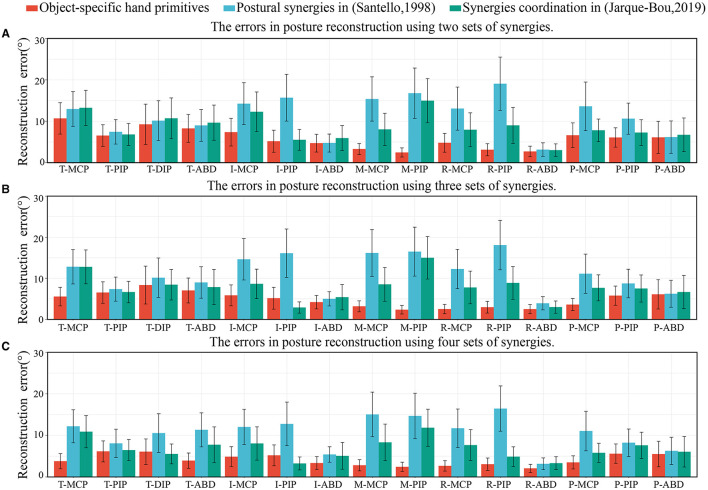
The errors in posture reconstruction when using two, three, and four sets of object-specific hand primitives and former postural synergies. **(A)** The errors in posture reconstruction using two sets of synergies. **(B)** The errors in posture reconstruction using three sets of synergies. **(C)** The errors in posture reconstruction using four sets of synergies.

The hand primitives proposed in this paper had a different function from the postural synergies extracted in previous studies—especially hand primitive 2. The second postural synergy in past work has usually been used to represent the opening and closing movements of the last three fingers with respect to right ones, or the flexion at the MCP joints with adduction in all fingers. However, that the inverse motion of the MCP and PIP joints is more important than the above postural synergies has rarely been considered. We compared the error in the reconstructed motion of the joints when using the hand primitives proposed in this study, and the postural synergies extracted by (Santello et al., 1998) and (Jarque-Bou et al., 2019). The errors in posture reconstruction when using two, three, and four sets of synergies are shown in Figure 13. The hand primitives proposed in this paper yielded good performance. When using two sets of synergies, the errors in the MCP and PIP joints of the middle and the ring fingers were significantly reduced comparing with the other two groups. When using three to four sets of hand primitives, error in the reconstruction of the thumb was reduced. However, using more postural synergies extracted by Santello et al. only slightly improved the accuracy of reconstruction, possibly because they considered only imaginary grasping tasks, which cannot accurately simulate empirical contact conditions. The error of posture reconstruction using four sets of synergies extracted by Jarque-Bou et al. also achieved a better result, but the movement of the MCP joints were still not ideal.

Functional verification experiments on a prosthetic hand were performed to evaluate our approach. The hand used in the experiment was linkage-driven, and the last four fingers were modular-designed. Each finger could provide two active DOFs (MCP and PIP joints) and one passive DOF (DIP joint). The thumb could also provide two active DOFs (MCP and ABD joints) and one passive DOF (IP joint). Besides, adaptive movement could be carried out between finger joints. The hand primitives were simplified according to the DOF-configuration of the hand. Primitive 1 controlled the coordinated closure of all fingers, primitive 2 controlled the PIP flexion of four fingers when the MCP joints were blocked, primitive 3 controlled the closing movements of the middle, ring, and little fingers with respect to the index finger, and primitive 4 controlled the adduction of the thumb.

**Figure 14:**
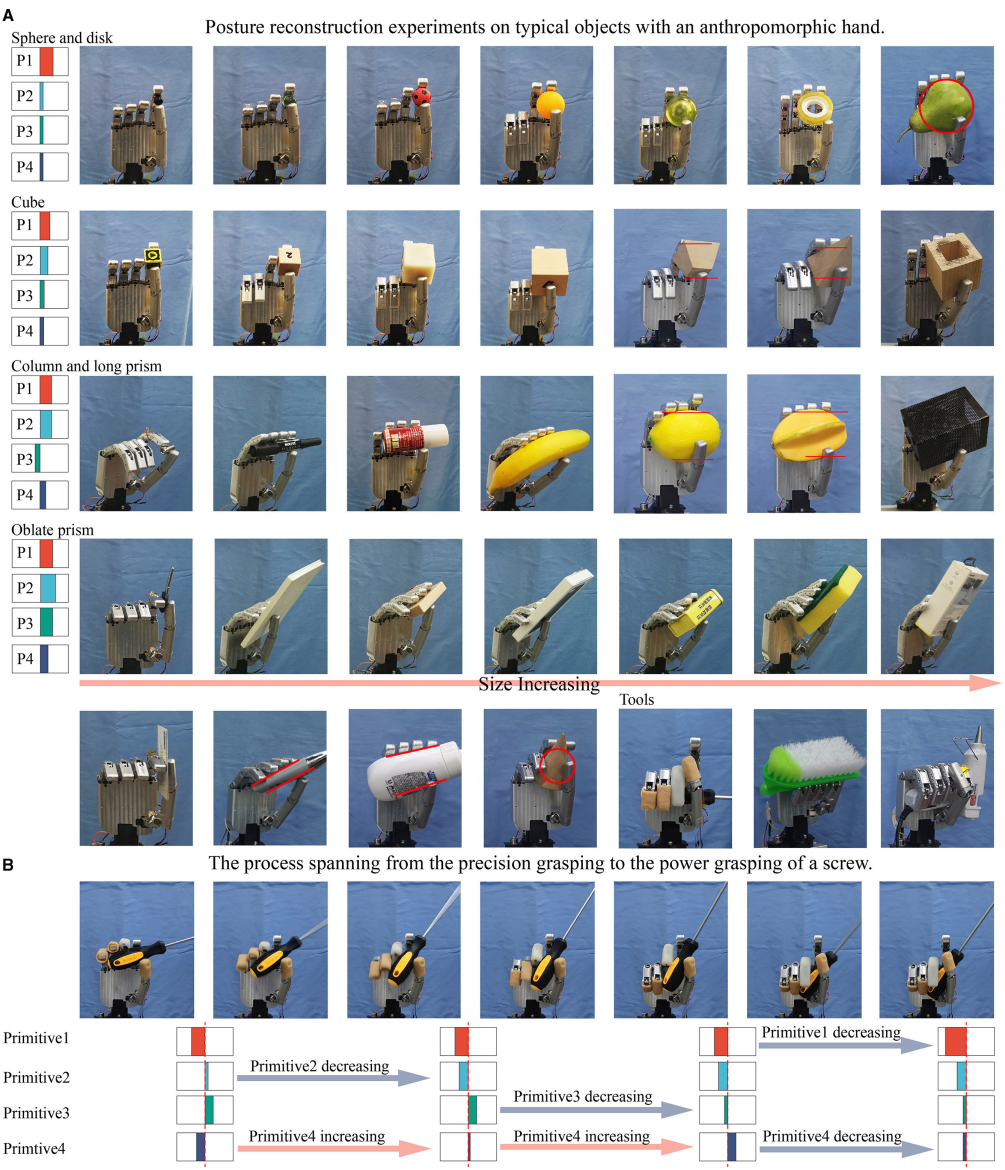
**(A)** The posture reconstruction experiments on a prosthetic hand, and the average synergy inputs of the hand primitives in different types of grasping tasks were shown. **(B)** The process spanning from the precision grasping to the power grasping of a screwdriver, and the tendency of four synergy inputs variation during the task was shown.

The performance in terms of posture reconstruction on typical objects is shown in Figure 14A. For each category, objects of increasing size were evaluated. Qualitatively, with the increase of object size, the number of fingers involved in grasping gradually increased. The postural configuration for a sample object was determined by the weighted mean and co-variance relative to reference trajectory in the corresponding category. While for irregular shaped objects, appropriate grasping strategies were selected according to the shape of their convex hull. For example, pear used grasping strategy of spherical objects, carambola used grasping strategy of prolate objects, and cleaner used grasping strategy of oblate objects. The results were generally consistent with the grasping strategies of the synergy learner, and the additional adaptive movement helped in closing the fingers until contact.

As discussed above, the biggest difference between grasping tools and other objects was that tools usually needed to adopt power grasping for further operation tasks. Therefore, when grasping the screwdriver, brush, and glue gun, we used a larger primitive 1 value to make each finger bend significantly. In addition, we also reproduced the process spanning from precision grasping to power grasping of a screwdriver as shown in Figure 14B. This complex task can be considered a combination of several groups of simple tasks. At the beginning, the screwdriver was pinched up using the grasping strategy of prolate objects. Then, through varying the value of primitives 2 and 4, the relative position of the thumb and four fingers were adjusted so that the object rotated counterclockwise. Next, the value of primitive 3 decreased and the primitive 4 increased, led to the thumb and index finger were released while the last three fingers held the object. Finally, the value of primitives 1 and 4 were reduced to enable the stable enveloping of the object by all fingers. This process was similar to the functionally divided manipulation synergy proposed in (Todorov and Ghahramani, 2004; Santina et al., 2019). Through this example, it can be seen that the four hand primitives extracted in this paper have certain in-hand postural adjustment ability, and can meet the requirements of complex operation tasks.

## Discussion

The experimental paradigm used in this paper included grasping and moving tasks involving five typical categories of objects as well as simulated tasks involving common tools. In previous studies, postures in the taxonomy of grasping have been set as the experimental paradigm. However, this taxonomy is based on the number of fingers involved in the task, because of which it contains many postures that are similar to one another but are not frequently used in daily life (like prismatic 2 finger, prismatic 3 finger, prismatic 4 finger). The frequency of picking up objects through precision grasping is higher in daily life than that through power grasping. Therefore, the experimental paradigm considered here can better fit human grasping habits, simplify postures in the grasping taxonomy, and help better understand grasping strategies from a data-driven perspective. Moreover, the size-related information of the objects was recorded, and can be used to identify the relationship between the trajectories of the synergy inputs and the grasped objects. In previous studies, this content has often been ignored even though it is important for automatic posture planning.

Because of the use of different experimental paradigms, the functions for the hand primitives extracted in this paper were different from those in previous studies. The most significant difference was in the primitive 2, which controlled the reverse movement of the MCP and PIP joints of the last four fingers. The most important effect of primitive 2 was to adjust the angle between the distal phalanx and the object. This conclusion is consistent with the perspective of anatomy. The musculoskeletal system of the human hand is complex. The flexor digitorum profundus (FDP) and superficialis (FDS) are the extrinsic flexors, radial interosseus (RI), ulnar interosseus (UI), and lumbrical (LU) are the intrinsic muscles, and the long extensor (LE) lumps the two extrinsic extensor muscles: the extensor digitorum communis (EDC) and extensor indicis (EI). Synek compared the maximum isometric fingertip based on the wEM model and the noEM model (Synek and Pahr, 2016). The results showed that the wEM model accords with the biomechanical properties of human hands. In this model, the routing of the extensor tendon crosses all joints of the fingers, and causes the flexion of the MCP joint as well as the extension of the PIP and DIP joints. This extensor can generate fingertip forces over a wide range of postures along several directions of force, and enables the finger to be more versatile during grasping. This form of movement is in accordance with primitive 2 in this paper. Implementing a similar structure for the anthropomorphic hands to improve their operational performance is a matter suitable for future investigation.

This study has some limitations. In the reconstruction experiment, not all fingers came into contact with the object, and the grasping forces exerted by different fingers were different. This shows that different fingers have varying importance for the success of grasping actions. Reasonably distributing the importance of different fingers in the relevant model, and ensuring that the more important joints come into contact with the object are important issues to advance investigation in this domain. In addition, although the flexion of the DIP joints could not be accurately calculated here, they play an important role in the adaptive movement. Data on them should thus be collected.

## Conclusion

This study proposed a method for predicting hand movements to grasp objects of different sizes. To acquire the necessary movement-related data, the angular values of the joints of 10 subjects were recorded as they grasped and moved typical objects in experiments and virtual operation tasks. Four hand primitives were extracted by PCA, and the corresponding trajectories of the synergy inputs during each task were saved. The Gaussian process was used to obtain the latent representation of the grasps and the diameter of the grasped object. Finally, hand movements were generated according to the distribution of grasp configurations for a given diameter of object and grasp type.

In the experimental paradigm used, the natural grasping habits of humans were considered, and the results showed that the frequency of precision grasping was much higher than that of power grasping. Only in operational tasks was power grasping required. The four hand primitives extracted here explained 87% of the variance. The function of primitive 2 was found to be significantly different from that in previous studies. It was important for adjusting the angle between the distal phalanx and the object, and for allowing the fingers to be more flexible. The results showed that the proposed method significantly reduced the error in the reconstructed posture, especially that in the MCP and PIP joints of the middle and ring fingers. Finally, we used a shape descriptor to infer the trajectories of the synergy inputs and plan the motion of the hand. The proposed, simplified hand primitives are useful for controlling the grasping movement of a prosthetic hand, and the relevant operational tasks can be reconstructed through segmentation.

This paper mainly explored the grasp planning of the typical shape objects. However, the grasping of complex objects by human hand can also be regarded as the local adaptive movement after the approximate posture configuration. Therefore, we will further analyze which joints the adaptive movement mainly occurs in, and reproduce adaptive movement with hand primitives through mechanical mechanism. Besides, in addition to the posture configuration, the distribution of grasping force also has a great influence on the success rate of grasping. In the future study, we will consider motion and mechanics data synthetically, evaluate the importance of each fingers to the grasping task, and give the stably grasping condition from the data-driven point of view.

## Data Availability Statement

The raw data supporting the conclusions of this article will be made available by the authors, without undue reservation.

## Ethics Statement

The studies involving human participants were reviewed and approved by Institutional Review Board (IRB) of the Harbin Institute of Technology in P. R. China. The patients/participants provided their written informed consent to participate in this study. Written informed consent was obtained from the individual(s) for the publication of any potentially identifiable images or data included in this article.

## Author Contributions

BL developed the idea, researched the literature, and contributed to the acquisition and analysis of the data. JD contributed to prosthetic hand experiment. LJ and SF revised the work. All authors contributed to the article and approved the submitted version.

## Funding

This work was supported in part by the China National Key Research and Development Program under Grant No.2020YFC2007801 and in part by the National Natural Science Foundation of China under Grant No. U1813209 and No. 91948302.

## Conflict of Interest

The authors declare that the research was conducted in the absence of any commercial or financial relationships that could be construed as a potential conflict of interest.

## Publisher's Note

All claims expressed in this article are solely those of the authors and do not necessarily represent those of their affiliated organizations, or those of the publisher, the editors and the reviewers. Any product that may be evaluated in this article, or claim that may be made by its manufacturer, is not guaranteed or endorsed by the publisher.
