# Supplementary material for: Learning Grasp Configuration Through Object-Specific Hand Primitives for Posture Planning of Anthropomorphic Hands
Source: Front Neurorobot. 2021 Sep 15;15:740262. doi: 10.3389/fnbot.2021.740262 (PMC8480411; doi:10.3389/fnbot.2021.740262)
Supplement: Supplementary file 1 [file Table_1.docx]

**APPENDIX**

Table Ⅰ. The dimensions of selected five categories of typical objects and a set of complex tools in Figure.3.

| Object | A | B | H | Virtual operation tasks |
| --- | --- | --- | --- | --- |
| Sphere01 | 13.81 | 13.81 | 13.81 |  |
| Sphere02 | 19.69 | 19.69 | 19.69 |  |
| Sphere03 | 25.35 | 25.35 | 25.35 |  |
| Sphere04 | 39.20 | 39.20 | 39.20 |  |
| Sphere05 | 48.03 | 48.03 | 48.03 |  |
| Sphere06 | 63.70 | 63.70 | 63.70 |  |
| Sphere07 | 75.60 | 78.90 | 79.90 |  |
| Sphere08 | 71.76 | 82.60 | 84.10 |  |
| Sphere09 | 74.38 | 82.47 | 88.08 |  |
| Cube01 | 22.48 | 22.48 | 22.48 |  |
| Cube02 | 29.47 | 29.47 | 29.47 |  |
| Cube03 | 40.15 | 40.15 | 40.15 |  |
| Cube04 | 48.95 | 48.95 | 48.95 |  |
| Cube05 | 58.00 | 58.00 | 58.00 |  |
| Cube06 | 71.99 | 71.99 | 109.29 |  |
| Cube07 | 23.50 | 23.50 | 75.10 |  |
| Cube08 | 28.55 | 29.28 | 59.55 |  |
| Cube09 | 40.00 | 40.00 | 145.00 |  |
| Column01 | 5.03 | 5.03 | 11.80 |  |
| Column02 | 13.90 | 13.90 | 50.46 |  |
| Column03 | 9.68 | 9.68 | 146.67 |  |
| Column04 | 18.56 | 18.56 | 145.81 |  |
| Column05 | 24.08 | 24.08 | 92.58 |  |
| Column06 | 35.00 | 35.00 | 100.00 |  |
| Column07 | 38.80 | 39.02 | 200.00 |  |
| Column08 | 60.00 | 60.00 | 100.00 |  |
| Column09 | 78.00 | 78.00 | 180.00 |  |
| Disk01 | 24.99 | 24.99 | 1.85 |  |
| Disk02 | 53.24 | 53.24 | 13.00 |  |
| Disk03 | 76.88 | 76.88 | 18.68 |  |
| Disk04 | 86.96 | 86.96 | 18.00 |  |
| Disk05 | 120.00 | 120.00 | 1.25 |  |
| Prism01 | 27.95 | 80.05 | 6.45 |  |
| Prism02 | 53.90 | 85.75 | 0.40 |  |
| Prism03 | 28.84 | 89.93 | 14.48 |  |
| Prism04 | 62.13 | 89.10 | 37.23 |  |
| Prism05 | 64.95 | 100.85 | 24.45 |  |
| Prism06 | 88.64 | 151.95 | 17.92 |  |
| Prism07 | 81.40 | 141.84 | 38.68 |  |
| Prism08 | 128.48 | 154.00 | 7.87 |  |
| Facial Cleanser | 33.00 | 33.00 | 150.37 | Prepare to squeeze |
| Spoon | 8.28 | 32.18 | 160.00 | Prepare to spoon up |
| Small screw driver | 20.23 | 20.23 | 131.18 | Prepare to screw |
| Scissors | 5.95 | 59.88 | 168.00 | Prepare to cut |
| Hammer | 25.47 | 40.63 | 161.84 | Prepare to hit the nail |
| Pliers | 17.84 | 57.53 | 156.90 | Prepare to clamp |
| Toothbrush | 6.20 | 10.24 | 182.00 | Prepare to brush |
| Comb | 3.64 | 17.94 | 184.00 | Prepare to comb |
| Wrench | 15.55 | 25.82 | 162.00 | Prepare to screw |
| Large screw driver | 27.98 | 27.98 | 187.00 | Prepare to screw |
